# Ruthenium-Based Defective MOFs as Heterogeneous Catalysts for the Hydrogenation of Carbon Dioxide to Formate

**DOI:** 10.1021/acssuschemeng.5c13124

**Published:** 2026-03-24

**Authors:** Shuting Li, Taizong Shen, Songqi Leng, Amarjeet Bassi, Dan Wu, Yining Huang, Chunbao Charles Xu

**Affiliations:** † Department of Chemistry, 6221 Western University, London, ON N6A 5B7, Canada; ‡ Department of Chemical and Biochemical Engineering, 6221 Western University, London, ON N6A 3K7, Canada; § School of Energy and Environment, 53025 City University of Hong Kong, Hong Kong 999077, Hong Kong

**Keywords:** MOF, formate, heterogeneous catalysis, carbon dioxide, hydrogenation

## Abstract

The hydrogenation of carbon dioxide to formic acid has attracted considerable interest for carbon utilization and hydrogen storage. Metal–organic frameworks (MOFs), featuring open coordination sites and tunable pore structures, offer unique advantages for heterogeneous catalysis. Herein, we report the catalytic performance of a series of defect-engineered Ru-MOF catalysts for the hydrogenation of CO_2_ to formate. The catalysts were synthesized by partially substituting the organic linker with 3,5-pyridinedicarboxylic acid (PYDC) to introduce controllable defect sites. Among the series, the defective Ru-MOF prepared with an optimal PYDC ratio of 30% used during synthesis (D3-Ru-MOF) exhibits enhanced catalytic performance for CO_2_ hydrogenation to formate compared with its nondefective counterpart. After hydrogen pretreatment, D3-Ru-MOF achieves a turnover number (TON) of 1258 at 120 °C over 24 h while maintaining good structural stability. A clear structure–activity relationship was observed with catalytic performance dependent on defect density within the framework. This study demonstrates the effectiveness of defect-engineering in Ru-MOFs for improving CO_2_ hydrogenation performance.

## Graphical abstract

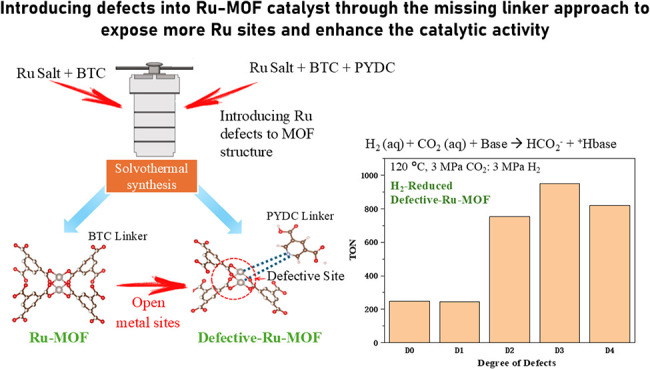

## 1 Introduction

The growth of the global population and the improvement of human living standards have significantly impacted world energy consumption.^1^ The effects of global warming are also accelerated by the carbon dioxide generated from human activities including transportation, chemical manufacturing, and fossil fuel combustion.^2^ Carbon dioxide is a common greenhouse gas. Research studying the conversion of carbon dioxide into other valuable resources has gained a lot of interest and provided solutions to the rising carbon dioxide issue. The utilization of carbon dioxide as feedstock for the fine chemical manufacturing can offer sustainable resources for human activities and contribute positively to mitigating global warming.^3^

Hydrogen, considered as a green energy source, has attracted great attention to be used in the future vehicle fuel systems and other diverse energy applications.^4^ Compared to traditional gasoline or diesel fuels, hydrogen provides the highest energy density by weight, producing only water as a byproduct.^5^ But the difficulty in converting hydrogen gas into a liquid as well as its flammable nature poses challenges in terms of transportation.^6^

CO_2_ to formic acid (HCOOH) provides solutions for the utilization of carbon dioxide and the transportation of hydrogen. Formic acid is a widely used fine chemical in the leather and textile industry. Also, formic acid can undergo dehydrogenation at mild conditions to produce hydrogen gas.^7^ The current H_2_ transportation involves compressing hydrogen gas at high pressure (200 bar) with a density of 16 g/L, while formic acid can achieve a higher hydrogen content of 53 g/L.^8^ Therefore, the use of formic acid as an organic liquid hydrogen carrier could provide a safe and scalable approach to hydrogen transportation and storage.

The hydrogenation of carbon dioxide to formic acid was initially reported by Farlow and Adkins in 1935, utilizing RANEY-nickel as a catalyst.^9^ In the 1970s, Inoue and coworkers published a report on carbon dioxide hydrogenation to formate salt, employing various transition-metal complexes (including Pd, Fe, Co, Ni, Ru, Rh, and Ir) under relatively mild conditions.^10^ Typically, the reaction between H_2_ and CO_2_ to form liquid formic acid is thermodynamically unfavorable (Δ*G*° = +33 kJ/mol) under standard conditions.^11^ To overcome the unfavorable thermodynamics, the aqueous phase and the presence of a base are normally employed with the generation of formate salts, which favor the thermodynamics of the process (Δ*G*° = −35 kJ/mol).^12^ The catalytic hydrogenation of carbon dioxide to formate over metal catalysts generally proceeds via initial metal–hydride formation, followed by CO_2_ adsorption and activation, generation of a metal–formate (OCHO) intermediate, and subsequent dissociation of formate, as illustrated in Reaction Scheme 1.^13^

**Scheme 1:**
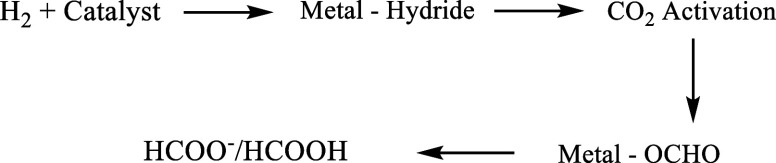
Metal–Hydride Mediated CO_2_ Hydrogenation to Formate

Over the past few decades, both homogeneous and heterogeneous catalytic systems have been investigated for the hydrogenation of carbon dioxide to formate. Supported transition-metal catalysts are widely employed in these reactions due to their good activity and selectivity toward formic acid/formate production.^2^ The first reported study on the CO_2_ hydrogenation reaction using first-row transition metals was conducted in 1975.^10^ In the homogeneous research, one 2012 research work used cobalt dihydride as a catalyst and obtained a TON of 3877.^14^ Another study in 2016 using an iron catalyst demonstrated a TON of 10275.^15^ With the introduction of a noble metal such as Ru, one study published in 2013 reached a TON of 23000.^16^

Homogeneous catalysts generally exhibit higher catalytic activity for formate production compared to heterogeneous catalysts.^17^ However, homogeneous catalysts suffer from high cost, difficulty in separation and limited recyclability.^18^ In contrast, heterogeneous catalysts offer advantages in easy separation, catalyst recovery, and recyclability.^19^ The field of heterogeneous CO_2_ hydrogenation has gained increasing attention in recent years. In 2017, Huang et al. reported a TON of 1026 using a gold catalyst immobilized on silica support.^20^ One study in 2022 demonstrated a TON of 7550 and a formic acid concentration of 0.355 M using a Ru catalyst dispersed on carbon hollow spheres.^21^

Metal–Organic Frameworks (MOFs) are crystalline porous materials composed of metal nodes coordinated with organic ligands.^22^ Due to their structural diversity and tunable pore size, MOFs have found applications in a wide range of fields, including gas storage and separation, catalysis, drug delivery, and chemical sensing.^23^ The structural stability, catalytic activity, and tunable pore size of MOFs make them highly promising as support materials for catalytic reactions.^24^ Although MOFs do not possess a well-defined molecular quadrupole moment, the local electrostatic fields and π-electron-rich aromatic linkers within MOF pores can interact favorably with the quadrupole moment of CO_2_, facilitating its adsorption and enrichment.^25^ The MOFs contain both hydrophobic and hydrophilic domains. The surface hydrophilic/hydrophobic properties of the catalyst also play an important role in reactant transport and reaction intermediate (formate) stabilization. Hydrophilic or polar surface environments enhance solvent wetting^26^ and stabilize polar intermediates.^27^ The existence of hydrophobic domains on catalyst surfaces can improve local CO_2_ enrichment near active sites, supporting CO_2_ adsorption and maintaining efficient heterogeneous behavior.^28^

Several research groups have explored the hydrogenation of carbon dioxide by using MOFs as heterogeneous catalysts. In these studies, MOFs generally serve as supporting materials, and the active transition-metal species are anchored onto the MOF structure through either impregnation or coordination with functional groups on the organic linkers. The reported TON values for these MOF-based catalytic systems range widely.^29–31^ Although MOFs have been extensively investigated as catalyst supports, studies employing MOFs themselves as the active catalysts for the hydrogenation of carbon dioxide to formic acid and formate remain relatively uncommon. Therefore, further efforts still need to be made to take advantage of the structural features of MOFs and thereby enhance the performance of MOF-based catalysts for CO_2_ hydrogenation.

Inspired by a previous study on defect-engineered Ru-MOFs reported in 2014 for gas sorption and hydrogenation reactions,^33^ we employed a series of Ru-MOFs synthesized using benzene-1,3,5-tricarboxylic acid (BTC) and 3,5-pyridinedicarboxylic acid (PYDC) as organic ligands focusing on extending this platform to CO_2_ hydrogenation to formate. The partial incorporation of PYDC introduced uncoordinated defect sites within the MOF framework, enabling the systematic modulation of defect density. Unlike Ru catalysts supported on N-doped carbon, where nitrogen primarily serves as an anchoring and electronic stabilization site, the utilization of nitrogen-containing linkers within an ordered MOF framework enables tuning of the local coordination environment and pore structure around the Ru centers. As a result, the defective Ru-MOFs exhibited enhanced catalytic performance in CO_2_ hydrogenation to formic acid, achieving a TON of 1258. As summarized in Table 1, previously reported Ru-based heterogeneous catalysts operate under different reaction systems and exhibit a broad range of TON values. Given that MOF-based heterogeneous catalysts for this reaction display a wide range of activities depending on temperature and pressure, this work demonstrates that the defective Ru-MOF catalyst performs competitively relative to other Ru-based systems when evaluated under similar reaction environments and pressures.^29^

**Table 1:** Comparison of Ru-Based Heterogeneous Catalysts for the Hydrogenation of CO_2_ to Formate/Formic Acid under Basic Conditions

Entry	Catalyst	Base	Solvent	TON
1	Mtz-RuCl_3_ ^32^	Et_3_N	EtOH	124
2	RuCl_3_@ZIF-8 ^32^	Et_3_N	EtOH	205
3	RuCl_3_@ZIF-8-Mtz (0.29)^32^	Et_3_N	H_2_O/EtOH	1008
4	RuCl_3_@MIL-101(Cr)-DPPB^29^	Et_3_N	DMSO/H_2_O/PPh_3_	831

## 2 Materials and Methods

### 2.1 Chemicals

Lithium chloride ≥99%, benzene-1,3,5-tricarboxylic acid (BTC) 98%, acetic acid, and acetic anhydride 99.5% were purchased from Sigma-Aldrich and used without further purification. Ruthenium­(III) chloride 98% and 3,5-pyridinedicarboxylic acid (PYDC) 98% were obtained from AA Blocks and used as received.

### 2.2 Synthesis of Catalysts

The Ru-precursor, D0-Ru-MOF, and the series of catalysts D1-Ru-MOF to D4-Ru-MOF were synthesized following a previously reported method developed by Fischer’s group.^33^

The Ru-precursor was synthesized based on a modified procedure. RuCl_3_·xH_2_O (0.5 g), LiCl (0.5 g), and acetic anhydride (3.5 mL) were added to glacial acetic acid (17.5 mL) in a 50 mL round-bottom flask. The mixture was stirred and refluxed at 150 °C for 24 h. The mixture was cooled down to R.T. (Room Temperature) and filtered. Then the resulting brown powder was washed using acetone.

D0-Ru-MOF was synthesized using a Ru precursor (170.0 mg) and BTC (100.0 mg). Both materials were mixed in 4 mL H_2_O and 0.7 mL of glacial acetic acid. The mixture was transferred into a stainless-steel Teflon-lined autoclave and heated to 150 °C for 48 h. The resulting dark green powder was collected by centrifugation and washed with water.

D1/D2/D3/D4-Ru-MOF was synthesized by adding different amounts of PYDC linkers during the synthesis procedure. D1-Ru-MOF: Ru-precursor (170.0 mg), BTC (96.0 mg), PYDC (4.0 mg); D2-Ru-MOF: Ru-precursor (170.0 mg), BTC (90.7 mg), PYDC (8.0 mg); D3-Ru-MOF: Ru-precursor (170.0 mg), BTC (70.5 mg), PYDC (24.0 mg); D4-Ru-MOF: Ru-precursor (170.0 mg), BTC (50.4 mg), PYDC (40.0 mg). The mixture was then combined with H_2_O (4.0 mL), acetic acid (0.7 mL) and transferred into a stainless-steel Teflon-lined autoclave and heated at 150 °C for 48 h. The mixture was collected through centrifugation and washed with water.

The D0-Ru-MOF and D1/D2/D3/D4-Ru-MOF were activated by heating at 150 °C for 24 h under a dynamic vacuum.

### 2.3 Characterization of Catalysts

Nitrogen adsorption–desorption isotherm experiments were performed using a Micromeritics ASAP 2020 surface area and porosity analyzer. Surface areas were calculated using the Brunauer–Emmett–Teller (BET) method. FTIR spectra were collected in the range of 550–4000 cm^–1^ using a PerkinElmer FTIR spectrometer. Hydrogen pretreatment of the catalyst was performed using a Quantachrome ChemBET TPR system for 5 h at 150 °C. The Ru content in the catalysts was quantified using an Agilent 7700 series ICP-MS system coupled with an Agilent 1260 Infinity HPLC. Liquid-phase ^1^H NMR spectra were recorded on a Bruker AVIII HD 400 MHz spectrometer. PXRD patterns were obtained by using a Rigaku SmartLab diffractometer equipped with Cu Kα radiation. Data were collected in the 2θ range of 5–45° with a step size of 0.01° and a scanning rate of 4°/min. Morphology and elemental mapping were conducted using Zeiss 1530 e-beam Lithography and Zeiss 1540XB FIB lithography. Samples were coated with a 3 nm osmium layer by using a Filgen Osmium Plasma Coater. XPS measurements were carried out using a Kratos AXIS Supra spectrometer to determine the oxidation states of Ru and N on the catalyst surfaces. TGA measurements were performed on a Mettler Toledo TGA 2 using 4–5 mg of sample heated from 30 to 600 °C at 10 °C min^–1^ under N_2_ (30 mL min^–1^).

### 2.4 Catalytic Hydrogenation of CO_2_ to Formate

All catalysts were analyzed before the catalytic reaction using Powder X-ray Diffraction (PXRD) to confirm structure. In a typical catalytic reaction, 5.0 mg of catalyst was introduced into a 15.0 mL reactor equipped with a magnetic stirrer. Subsequently, a total of 9.0 mL of solvent and 5 mmol of KOH were added to the reactor. The mixture was pressurized to a total pressure of 60 bar of carbon dioxide and hydrogen gas. The reaction mixture was heated to 120 °C for 24 h. The quantity of formate in the product mixture was determined using ^1^H NMR, with DMF as an internal standard and deuterium oxide as the solvent. The amount of Ru was determined using ICP-MS to calculate the TON (turnover number) and TOF (turnover frequency). All catalysts were collected by centrifugation and dried at 80 °C.

To evaluate the reusability of the catalysts, the solid catalyst was isolated after each reaction by centrifugation, followed by thorough washing several times with deionized water to remove residual reactants and products. The recovered catalyst was then oven-dried prior to reuse. For H2D3-Ru-MOF, the recovered catalyst was subjected to a hydrogen pretreatment before each subsequent catalytic experiment to restore the active sites.

## 3 Results and Discussion

### 3.1 Structure Characterization

Defective Ru-MOFs were synthesized using a mixed-linker strategy to introduce controlled structural defects into the framework. 3,5-Pyridinedicarboxylic acid (PYDC) was partially substituted for benzene-1,3,5-tricarboxylic acid (BTC) during synthesis to generate missing-linker defects, with the defect density increasing as the PYDC content increased (Figure 1a). The PYDC substitution levels (5–50%), defined as the fraction of BTC replaced by PYDC in the synthesis mixture, were chosen to represent low, moderate, and excessive defect levels. Because PYDC is a ditopic linker, whereas BTC is tritopic, these values are not directly comparable to BTC content in the parent framework but instead reflect controlled reduction of framework connectivity.^34^ Low PYDC content introduces low-density defect sites, whereas moderate levels (20–30%) maximize defect density and changes in the electronic state of Ru centers while maintaining framework integrity. In contrast, a high PYDC fraction (50%) exceeds the structural tolerance of the framework and illustrates the effects of excessive defect incorporation.

**Figure 1:**
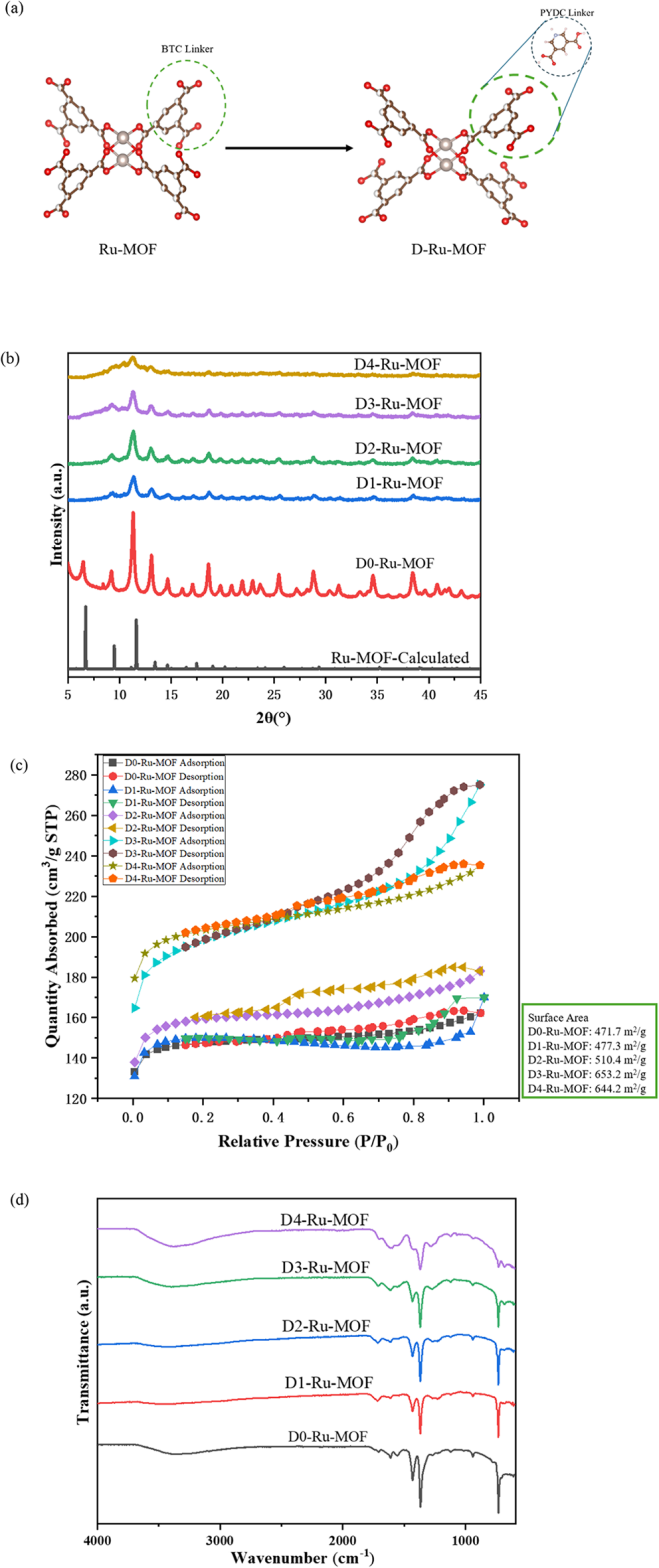
(a) Comparison between the linker used for D0-Ru-MOF and defective Ru-MOF;^38^ (b) PXRD patterns of D0-Ru-MOF and defective D1/D2/D3/D4-Ru-MOF catalysts prepared by adding different amounts of PYDC during the synthesis; (c) Nitrogen adsorption–desorption isotherms of defective D0/D1/D2/D3/D4-Ru-MOF catalysts with varying defect levels; (d) Fourier Transform Infrared spectrum of as-made D0/D1/D2/D3/D4-Ru-MOF.

The structure of the synthesized Ru-MOF and defective Ru-MOFs was confirmed by powder X-ray diffraction (PXRD) in the 2θ range between 5° and 45° (Figure 1b). The experimental PXRD pattern of Ru-MOF closely matches with the calculated pattern.^35^ This consistency suggests the successful formation of the desired framework. The sharp and well-defined peaks observed in the as-made sample reflect the high crystallinity of the experimental material. The positions of the major peaks are in good agreement with the calculated pattern, confirming that the as-made sample adopts the expected Ru-MOF structure. Some peaks are slightly shifted and located at different positions such as the peak around 25°; this might be due to the solvent remaining within the MOF pores. The presence of long-range ordered guest molecules can influence the PXRD peak positions by altering the local environment within the framework.^36^ D1-Ru-MOF to D4-Ru-MOF represent 5%, 20%, 30%, and 50% PYDC linker added during the synthesis. As shown in Figure 1b, the majority of peaks for D1-Ru-MOF, D2-Ru-MOF, and D3-Ru-MOF are closely matched with D0-Ru-MOF, the intensities for these three patterns are similar and reduced by almost half compared to the defect-free D0-Ru-MOF. This indicates that the overall lattice parameters and crystal symmetry are maintained, but the structural defects introduced during synthesis cause the loss of a long-range ordered crystal lattice.^37^ For D4-Ru-MOF, the weakened peak features and reduced intensity indicate that the structure of D4-Ru-MOF starts to lose crystallinity and becomes partially amorphous.

The nitrogen adsorption–desorption isotherms provided in Figure 1c illustrate the influence of structural defects on the porosity and surface area of the samples. Both D0-Ru-MOF (471.7 m^2^/g) and D1-Ru-MOF (477.3 m^2^/g) exhibit relatively low nitrogen uptake across the entire relative pressure range, suggesting a lower surface area and pore size. Introduction of a higher number of defects in D2-Ru-MOF (510.4 m^2^/g) by adding 20% PYDC during synthesis results in an increased nitrogen uptake, indicating a larger surface area compared to D0-Ru-MOF and D1-Ru-MOF. D3-Ru-MOF displays the highest nitrogen adsorption, which exhibits the highest pore volume and defects-rich framework. The gap formed between adsorption and desorption of D3-Ru-MOF confirms the presence of mesopores. The specific surface areas calculated by the BET method are 653.2 m^2^/g for the D3-Ru-MOF sample, which is the largest among the samples. Although the amount of PYDC used during synthesis for D4-Ru-MOF (644.2 m^2^/g) is the highest, its nitrogen uptake is lower than that of D3-Ru-MOF. This reduction is consistent with the PXRD results, which reveal partial structural collapse due to the excessive defect formation and lead to a loss of crystallinity.

The FTIR spectrum (Figure 1d) of the synthesized D-Ru-MOF displays vibration bands in the range of 4000–500 cm^–1^. The broad peak observed around 3300 cm^–1^ corresponds to −OH stretching, indicating the presence of solvent molecules (H_2_O). The sharp peaks between 1600 and 1400 cm^–1^ can be assigned to carboxylate groups (COO^–^) confirming coordination between the organic linkers (BTC and PYDC) and the metal center. Based on the FTIR results, there is not much difference among the samples, and the spectra do not indicate significant variation with different ratios of PYDC replacing BTC to create defects.

The XPS spectra (Figure 2a) display the Ru 3d and C 1s regions for samples D1-Ru-MOF to D4-Ru-MOF, with increasing defect concentrations introduced by partial substitution of the BTC linker with PYDC. The peak at 288.5 eV (green) is assigned to the C 1s signal from carboxylate groups (−COO), while the peak at 285.0 eV (red) corresponds to hydrocarbon C 1s. The peaks at approximately 286.7 eV (pink) and 282.5 eV (blue) are attributed to Ru­(II) species, and their intensity increases with higher PYDC content, which indicates a higher content of Ru­(II) sites present in the structure. Ru­(III) species are identified by peaks at 285.7 eV (gray) and 281.5 eV (light orange). The two peaks appearing in the D3-Ru-MOF and D4-Ru-MOF spectra at 284.2 eV (cyan) and 280.0 eV (orange) suggest the formation of partially reduced Ru^δ+^ species (0 < δ < 2), which are known to exhibit enhanced catalytic activity in CO_2_ hydrogenation.^39^ The decrease in the Ru^3+^ component (gray) accompanied by an increase in the Ru^2+^/Ru^δ^+^^ component (orange) from D1-Ru-MOF to D3-Ru-MOF indicates that the introduction of defect sites through PYDC incorporation induces a clear electronic state modulation of the Ru centers. This evolution reflects a partial reduction of Ru^3+^ to more electron-rich Ru^2+^/Ru^δ^+^^ species. The XPS results indicate a systematic evolution in Ru electronic states with increasing defect incorporation, which is consistent with the electronic modulation of the Ru centers. The reduced Ru^δ^+^^ contribution in D4-Ru-MOF compared to D3-Ru-MOF is attributed to partial framework collapse induced by excessive defect incorporation, which disrupts the electronic stabilization of reduced Ru species.

**Figure 2:**
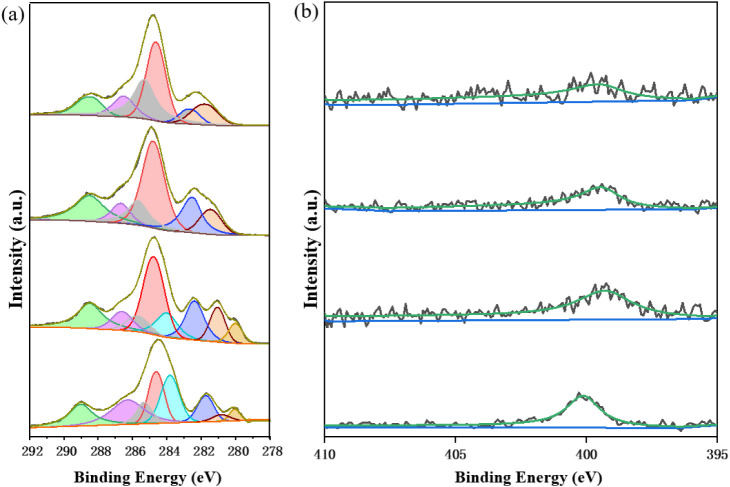
XPS spectra of defective Ru-MOF catalysts with varying defect levels (D1/D2/D3/D4-Ru-MOF, top to bottom); (a) C 1s and Ru 3d regions; (b) N 1s region.

Figure 2b spectra show the N 1s XPS of D1-Ru-MOF to D4-Ru-MOF, reflecting the increasing incorporation of the nitrogen-containing PYDC linker to the MOF structure. From top to bottom (D1-Ru-MOF to D4-Ru-MOF), an increase in signal intensity at ∼400 eV is observed. In D1-Ru-MOF, where only 5% PYDC is introduced during synthesis, the N 1s peak is weak and poorly defined. As the PYDC content increases from D2-Ru-MOF to D4-Ru-MOF, the N 1s signal becomes more intense and well-defined, confirming the successful incorporation of the nitrogen linker into the MOF structure. The increasing N 1s intensity is consistent with the formation of more nitrogen-associated defect sites, which may act as active sites and contribute to the enhancement of catalytic performance.

Thermogravimetric analysis (TGA) and derivative thermogravimetry (DTG) were employed to evaluate the thermal stability and quantitatively determine the linker composition of the defective Ru-MOF catalysts (D0/D1/D2/D3/D4-Ru-MOF), as shown in Figure S5 and Table S2. D1/D2/D3/D4-Ru-MOF samples exhibit three mass loss regions. The first weight loss step observed between 53 and 129 °C is attributed to the removal of physically adsorbed guest molecules and residual solvent trapped within the MOF pores. The second mass loss region (348–416 °C) corresponds to the decomposition of the PYDC linker, while the third region at higher temperatures (456–503 °C) is assigned to the decomposition of the BTC linker. The relative contents of guest molecules, PYDC, and BTC were calculated and are summarized in Table S2. As the PYDC content increases from D1-Ru-MOF to D3-Ru-MOF, a corresponding decrease in BTC contribution is observed, confirming successful mixed-linker incorporation and controlled defect introduction. In contrast, D4-Ru-MOF shows a reduced PYDC and BTC fraction, which is consistent with excessive defect incorporation and partial framework destabilization.

### 3.2 Performance of Ru-MOF and Defective-Ru-MOF Catalysts

A series of catalytic reactions were conducted to evaluate the activity of Ru-based materials under the same reaction conditions: 120 °C, a 1:1 CO_2_/H_2_ ratio (60 bar in total), KOH as the base, and methanol as the solvent as shown in Table 2. The catalytic performance for each catalyst was calculated based on the amount of formate produced and the corresponding TON. A control experiment conducted in the absence of a catalyst resulted in no detectable formate formation, confirming that the transformation does not proceed without a catalyst.

**Table 2:** Catalytic Performance of Ru-MOF Catalysts with Varying Defect Levels

Entry^a^	Catalyst^a^	mmol^b^	TON^b^,^c^
1	None	0	0
2	D0-Ru-MOF	1.07	247
3	D1-Ru-MOF	1.07	247
4	D2-Ru-MOF	3.19	735
5	D3-Ru-MOF	4.12	949
6	D4-Ru-MOF	3.56	820

a Reaction conditions: 5 mg catalyst, 9 mL MeOH, 5 mmol KOH, 120 °C, 24 h, 30 bar CO_2_ and 30 bar H_2_.

b The mmol of formate was calculated based on ^1^H NMR analysis using DMF as the internal standard.

c Loading was obtained from ICP-MS.

Entries 2 to 6 correspond to MOF-based heterogeneous catalysts with varying amounts of PYDC added during synthesis. D0-Ru-MOF and D1-Ru-MOF exhibited comparable TON values of 247 and 247, reflecting a minimal influence of the 5% PYDC incorporation in D1-Ru-MOF. An increase in activity was observed for D2-Ru-MOF (TON = 735), suggesting enhanced catalytic performance due to increased defect formation and more open Ru active sites. Among all samples, D3-Ru-MOF showed a relatively higher formate yield (4.12 mmol) and a TON of 949, which correlates with the well-defined crystallinity and optimal defect density in the structure. Although sample D4-Ru-MOF used the highest amount of PYDC to create defects during the synthesis procedure, it did not yield the highest TON. The PXRD analysis of D4-Ru-MOF indicated the partial structural collapse, resulting in a lower TON of 820. This observation suggests that D4-Ru-MOF might show structural instability for successive experiments. The initial catalytic investigation on defective Ru-MOF catalysts suggests that introducing 30% PYDC to replace BTC to create defects in the MOF structure leads to enhanced catalytic activity in the hydrogenation reaction.

The incorporation of PYDC linkers introduces pyridinic-N sites into the Ru-MOF framework, which creates polar microenvironments within the pores. Previous studies have shown that surface hydroxyl groups can stabilize formate species and modify reaction pathways by providing a polar environment at the catalyst interface.^40^ N-doped heterogeneous catalysts have been reported to enhance the hydrogenation of CO_2_ to formate. The N-containing functional group is one of the key factors to improve the catalytic performance.^41^ For a pyridinic-N site, it can interact weakly with CO_2_ through electrostatic and quadrupole interactions between the lone pair and the electron-deficient carbon center of CO_2_, increasing the local CO_2_ concentration without inducing strong chemisorption. This CO_2_ enrichment enhances catalytic performance by increasing the availability and residence time of CO_2_ near Ru active sites, increasing the probability of insertion of CO_2_ during formate formation.

### 3.3 Effects of Hydrogen Pretreatment of the Defective Ru-MOF Catalysts

To further evaluate the catalytic performance of different Ru-MOF catalysts for CO_2_ hydrogenation, the catalysts were subjected to hydrogen pretreatment prior to the reaction. According to Han et al., the catalytic cycle for CO_2_ hydrogenation to formate initiates with the insertion of CO_2_ into a Ru-hydride (Ru–H) species, highlighting the importance of hydride formation.^42^ Hydrogen pretreatment is expected to promote the formation of Ru–H species, which are widely considered to be key intermediates for catalyst activation and the establishment of the reactive state necessary for efficient CO_2_ hydrogenation.

Figure 3a presents the catalytic performance of H2D3-Ru-MOF (D3-Ru-MOF after Hydrogen Pretreatment) under varying pressures, and Figure 3b shows different pressure ratios of CO_2_/H_2_. In the pressure study, an increase in total pressure correlates with enhanced formate production. This improvement is attributed to the increased solubility of gaseous reactants (CO_2_) in the methanol solvent under higher pressure, which facilitates greater reactant availability in the liquid phase and reduces the activation barrier for hydrogenation. At 60 bar, the formate yield reaches its maximum and the H2D3-Ru-MOF catalyst maintains structural integrity during the reaction. The second graph in Figure 3b evaluates the influence of the CO_2_/H_2_ molar ratios on the formate yield under a constant total pressure of 60 bar. An increase in formate production is observed with higher hydrogen ratios, which suggests that excess hydrogen shifts the reaction equilibrium toward formate formation. Among the tested ratios, the 1:3 CO_2_/H_2_ ratio exhibits the most favorable performance and catalyst stability.

**Figure 3:**
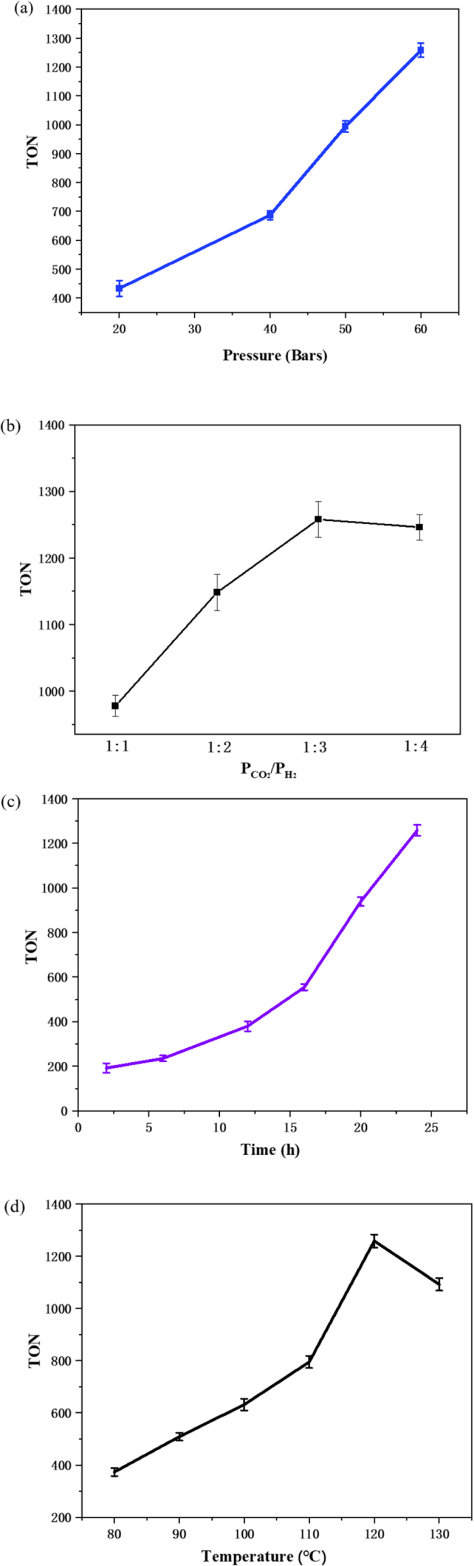
Comparison of the catalytic activity of the H2D3-Ru-MOF catalyst (a) under different total pressure of CO_2_ and H_2_ (1:1 v/v) (120 °C, 24 h); (b) under the same total pressure of 60 bar with different ratios of CO_2_/H_2_ (120 °C, 24 h); (c) for different lengths of reaction time (Total pressure of 60 bar, 120 °C); (d) at different temperatures (Total pressure of 60 bar, 24 h).

To further understand the catalytic behavior of the H2D3-Ru-MOF catalyst under different conditions, the effects of reaction time, temperature, and catalyst recyclability were systematically investigated. In Figure 3c, the formate yield is shown to increase gradually with reaction time, indicating that the H2D3-Ru-MOF catalyst maintains its catalytic activity throughout the duration of the reaction. The continuous increase in yield from 2 to 24 h suggests that the active sites within the catalyst framework remain stable and effective and do not show significant deactivation over time. Figure 3d illustrates the effect of reaction temperature on catalytic performance using the H2D3-Ru-MOF catalyst and evaluates performance at 80 °C, 90 °C, 100 °C, 110 °C, 120 °C, and 130 °C. An increase in formate yield is observed as the temperature rises from 80 °C to 120 °C, which indicates an enhancement in catalytic activity with higher temperature. The reaction achieves its maximum TON of 1258 at 120 °C. Beyond this temperature, a decline in formate yield is observed at 130 °C, which may be attributed to catalyst degradation induced by elevated temperature under strongly basic reaction conditions, as MOF frameworks are known to undergo structural instability in alkaline environments.^43,44^

The recyclability of the H2D3-Ru-MOF catalyst over four consecutive reaction cycles is shown in Figure 4. The TON decreases from 1258 in Run 1 to 1070 and 1085 in Runs 2 and 3 (∼15% decrease), indicating that partial deactivation begins before severe catalytic activity loss occurs. A more dramatic drop is observed in Run 4 suggesting a change in the dominant deactivation mechanism at later stages. To understand the structural evaluation of the catalyst during the reaction, the D3-Ru-MOF under hydrogen pretreatment and the used H2D3-Ru-MOF in a reusability test were evaluated by PXRD as shown in Figure 5a. The as-synthesized D3-Ru-MOF displayed distinct and sharp diffraction peaks, indicating a crystallized framework structure. After hydrogen pretreatment (H2D3-Ru-MOF), the PXRD pattern was retained and showed improved peak intensity and more well-defined peak positions, suggesting the hydrogen pretreatment process activated D3-Ru-MOF and removed solvent residue inside the structure pores. The stability of H2D3-Ru-MOF after the first catalytic cycle shows slightly decreased intensity accompanied by some structural collapse. Two large peaks formed at 38° and 42° correspond to the Ru nanoparticles’ hcp lattice.^45,46^ Metallic Ru diffraction peaks appear after the first reaction cycle, indicating the formation of Ru nanoparticles during catalysis.

**Figure 4:**
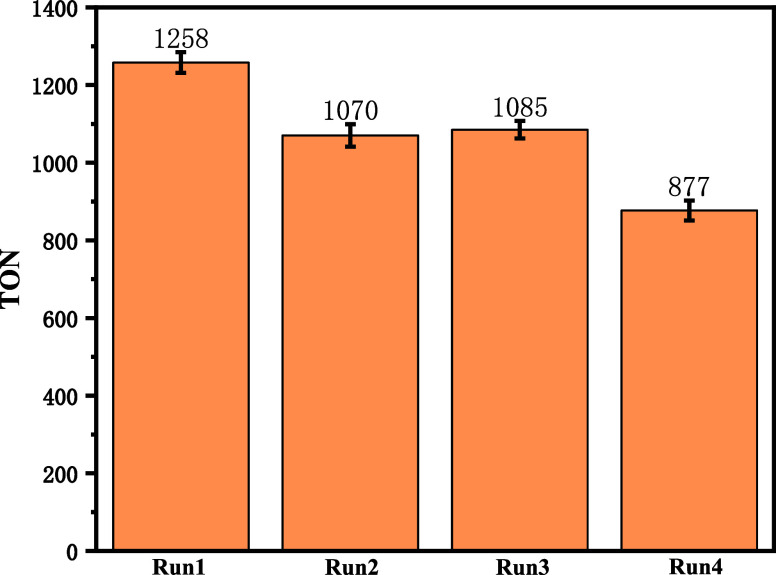
Recyclability tests of H2D3-Ru-MOF.

**Figure 5:**
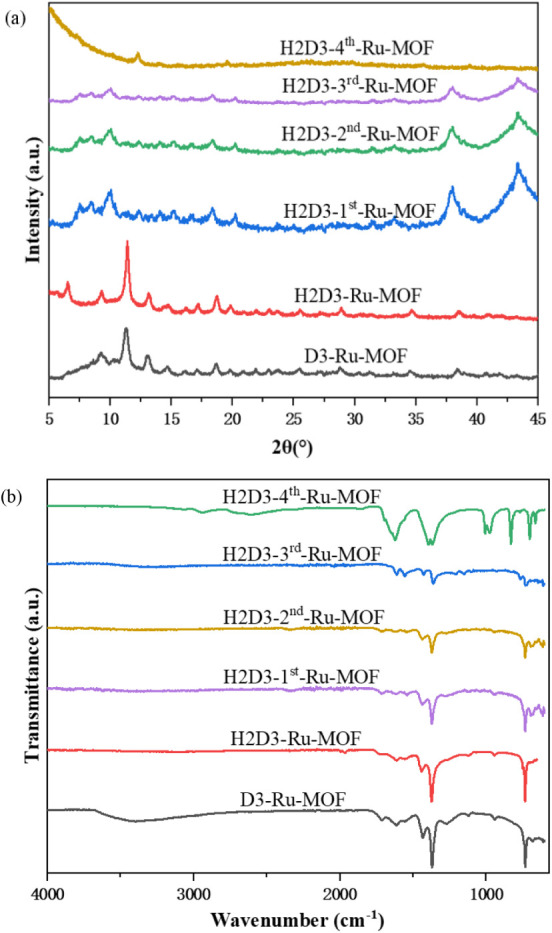
PXRD patterns (a) and FTIR spectra (b) of the D3-Ru-MOF catalyst before and after hydrogen pretreatment (H2D3-Ru-MOF) and the used H2D3-Ru-MOF after various cycles.

As the reaction progressed to the third and fourth cycles (H2D3-3rd-Ru-MOF and H2D3-4th-Ru-MOF), the PXRD patterns exhibited increasingly broadened and weakened peaks, indicating a loss of crystallinity. The gradual weakening and eventual disappearance of these reflections in subsequent cycles suggest progressive Ru leaching and loss of crystalline Ru domains rather than continued particle growth. Combined with the observed degradation of MOF crystallinity after the fourth cycle, these results indicate that catalyst deactivation evolves from Ru nanoparticle formation in the early cycles to framework loss in later cycles. This structural degradation is likely attributed to repeated exposure to hydrogenation conditions, leading to the accumulation of framework defects and the partial collapse of the MOF structure. The presence of residual diffraction peaks after the first and third cycles suggests that part of the crystalline framework was retained.

FTIR was employed to provide additional information in evaluating the structural stability of D3-Ru-MOF under various conditions, as presented in Figure 5b. The FTIR patterns of D3-Ru-MOF with hydrogen pretreatment, after the first and third reaction cycles, remained consistent with the as-synthesized sample. The peaks at approximately 1600 cm^–1^ and 1400 cm^–1^ correspond to the asymmetric and symmetric stretching vibrations of coordinated carboxylate groups from BTC and PYDC linkers. In the as-made D3-Ru-MOF sample, a broad band around 3300 cm^–1^ was observed, which is attributed to the stretching of the O–H bonds from residual solvent molecules. This band disappears after hydrogen pretreatment, suggesting successful solvent removal and catalyst activation. Two peaks appear around 2000 cm^–1^ in the H2D3-Ru-MOF sample, which confirms the successful formation of Ru–H species. Regarding the stability of the H2D3-Ru-MOF after the fourth reaction cycle, the FTIR spectrum exhibits significant peak broadening, and a significant change indicates alteration of the framework from the initial pattern. These changes provide evidence of structural degradation of the framework with repeated catalytic cycles.

ICP-MS analysis of Ru content (Table 3) shows Ru loading (wt %) in different Ru catalysts. The parent Ru-MOF with no defects and D1/D2/D3/D4-Ru-MOF show similar Ru amounts around 35 wt %. The Ru loading remained essentially unchanged after hydrogen pretreatment (35.34 → 35.10 wt %), indicating that the metal nodes were preserved without significant Ru leaching. However, the Ru content sharply decreased to 8.78 wt % after the fourth catalytic run, confirming the significant loss of Ru species. The dramatic Ru loss after Run 4 (∼75% loss) becomes the primary factor responsible for the substantial activity decline in the fourth cycle. This observation is consistent with the PXRD and FTIR results of the H2D3-4th-Ru-MOF sample, which demonstrate a clear collapse of the structural framework. The decrease in catalytic performance during the recyclability tests can be attributed to structural degradation associated with Ru loss, leading to a decreased number of active sites.

**Table 3:** Ru Contents of the Catalysts (Determined by ICP-MS)

Catalyst	Ru (wt %)
Ru-MOF	33.30
D1-Ru-MOF	34.75
D2-Ru-MOF	35.14
D4-Ru-MOF	36.89
D3-Ru-MOF	35.34
H2D3-Ru-MOF	35.10
H2D3-4th-Ru-MOF	8.78

The SEM images in Figure S6 illustrate the morphological evolution of the D3-Ru-MOF catalyst under three conditions: as-made sample, after hydrogen pretreatment sample, and after the fourth catalytic cycle sample. The as-made D3-Ru-MOF sample displays a well-defined, aggregated morphology characteristic of a porous structure with a high surface area and is suitable for catalytic reactions. Following hydrogen pretreatment, the H2D3-Ru-MOF sample retains most of its original morphology, indicating that the catalyst activation process does not significantly affect the structural integrity. However, after the fourth reaction cycle, the H2D3-Ru-MOF catalyst exhibits a significant morphological degradation. The surface appears to be less porous, and the overall structure shows features different from those of the original morphology, suggesting a collapse of the framework. This morphological change corresponds to the observed decrease in catalytic activity during the fourth run of the reusability tests.

## 4 Conclusions

In this study, a series of Ru-based MOF catalysts with controllable defect sites were evaluated for the hydrogenation of CO_2_ to formate. Incorporation of defects into the catalyst structure using PYDC significantly enhanced the catalytic performance by increasing the surface area, porosity, and accessibility of active Ru sites. Among the synthesized catalysts, D3-Ru-MOF with 30% PYDC added during synthesis exhibited the highest formate yield and TON, attributed to its optimal defect density and relatively stable crystallinity. Hydrogen pretreatment further improved catalytic activity by generating Ru–H species, which serve as a crucial intermediate for CO_2_ activation during catalysis. While XPS and FTIR provide indirect evidence, further in situ or operando studies would be valuable to directly verify hydride formation and electronic modulation of Ru during the reaction. Reusability tests indicated that while H2D3-Ru-MOF retained its activity for the first three cycles, significant structural degradation was observed after the fourth cycle. This work highlights the effectiveness of defect-engineering and catalyst pretreatment strategies in enhancing Ru-MOF performance for CO_2_ hydrogenation and provides insight for improving stability and catalytic efficiency through structural design and optimization of reaction conditions.

## Supplementary Material



## Notes

Additional studies on using Ru-MOFs as catalysts, TGA results, NMR data, and SEM images (PDF)
