# Supplementary material for: Ruthenium-Based Defective MOFs as Heterogeneous Catalysts for the Hydrogenation of Carbon Dioxide to Formate
Source: ACS Sustain Chem Eng. 2026 Mar 24;14(13):6355–64. doi: 10.1021/acssuschemeng.5c13124 (PMC13058891; doi:10.1021/acssuschemeng.5c13124)
Supplement: Supplementary file 1 [file sc5c13124_si_001.pdf]

Ruthenium-based Defective MOFs as Heterogeneous Catalysts for Hydrogenation of Carbon Dioxide to Formate

Shuting Li[a], Taizong Shen[b], Songqi Leng[b], Amarjeet Bassi[b], Dan Wu[c], Yining Huang[a]* and Chunbao Charles Xu[c]*

[a]Department of Chemistry, Western University, London, ON, N6A 5B7, Canada

[b]Department of Chemical and Biochemical Engineering, Western University, London, ON, N6A 3K7, Canada

[c]School of Energy and Environment, City University of Hong Kong, 999077, Hong Kong

*Corresponding authors: yhuang@uwo.ca (Y.H.) and chunbaxu@cityu.edu.hk (C.X.)

This file contents:

Number of figures: 8

Number of tables: 2

Page S3: **Fig. S1.** XRD patterns of the calculated and as-synthesized Ru-MOF.

Page S4: **Fig. S2.** SEM-EDX data for D3-Ru-MOF, H2D3-Ru-MOF and H2D3-3rd -Ru-MOF catalyst

Page S5: **Fig. S3.** ^{1}H NMR spectrum for the reaction product obtained with D2-Ru-MOF catalyst.

Page S6: **Fig. S4.** FTIR spectra of the D3-Ru-MOF catalyst before and after hydrogen treatment (H2D3-Ru-MOF)

Page S7: **Fig. S5.** Thermogravimetric analysis (TGA) and derivative thermogravimetry analysis (DTA) curves of D0/D1/D2/D3/D4-Ru-MOF

Page S8: **Fig. S6.** SEM images of D3-Ru-MOF catalysts

Page S9: **Fig. S7.** Effect of Solvent

Page S10: **Fig. S8.** Effect of Base

Page S11: **Table S1** Summary of results of studies using MOF as heterogeneous catalysts

Page S12: **Table S2** Composition of Defective Ru-MOF catalysts with varying PYDC/BTC ratios and solvent content

Page S13: References

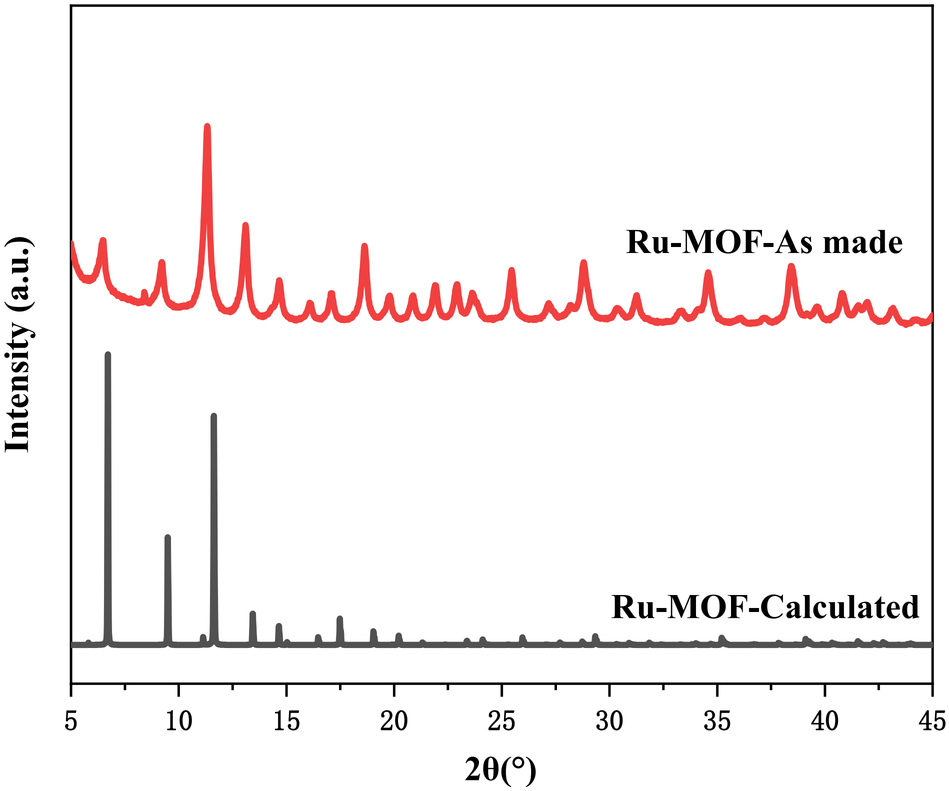

Figure S1. XRD patterns of the calculated and as-synthesized Ru-MOF.

(a)

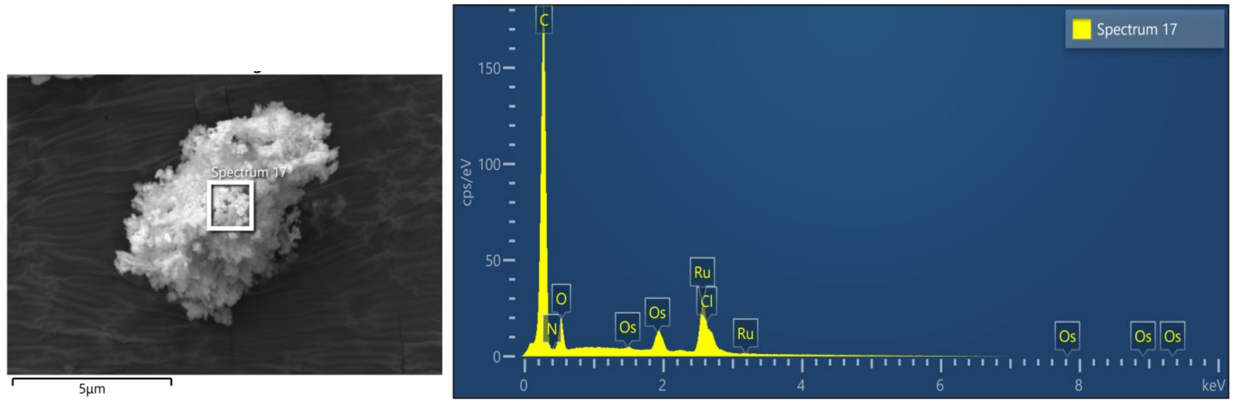

(b)

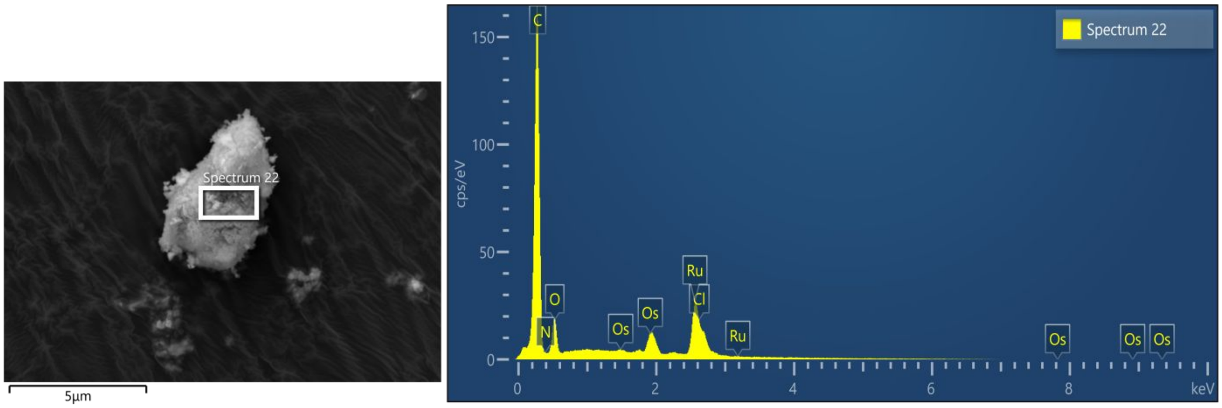

(c)

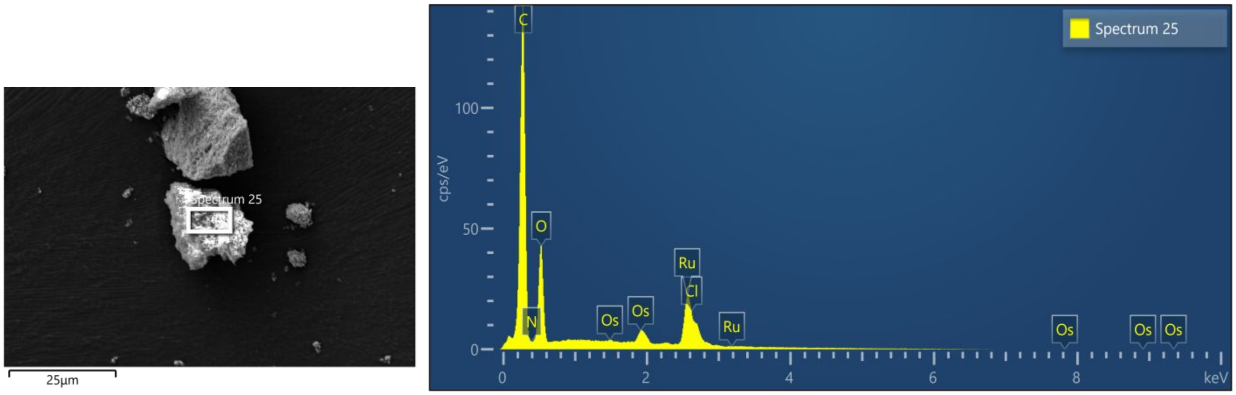

Figure S2. (a) SEM-EDX data acquired for D3-Ru-MOF catalyst, (b) SEM-EDX data acquired for H2D3-Ru-MOF catalyst, (c) SEM-EDX data acquired for H2D3-Ru-MOF catalyst after 3rd reaction cycle. The average atomic ratio of Ru is 8.5%.

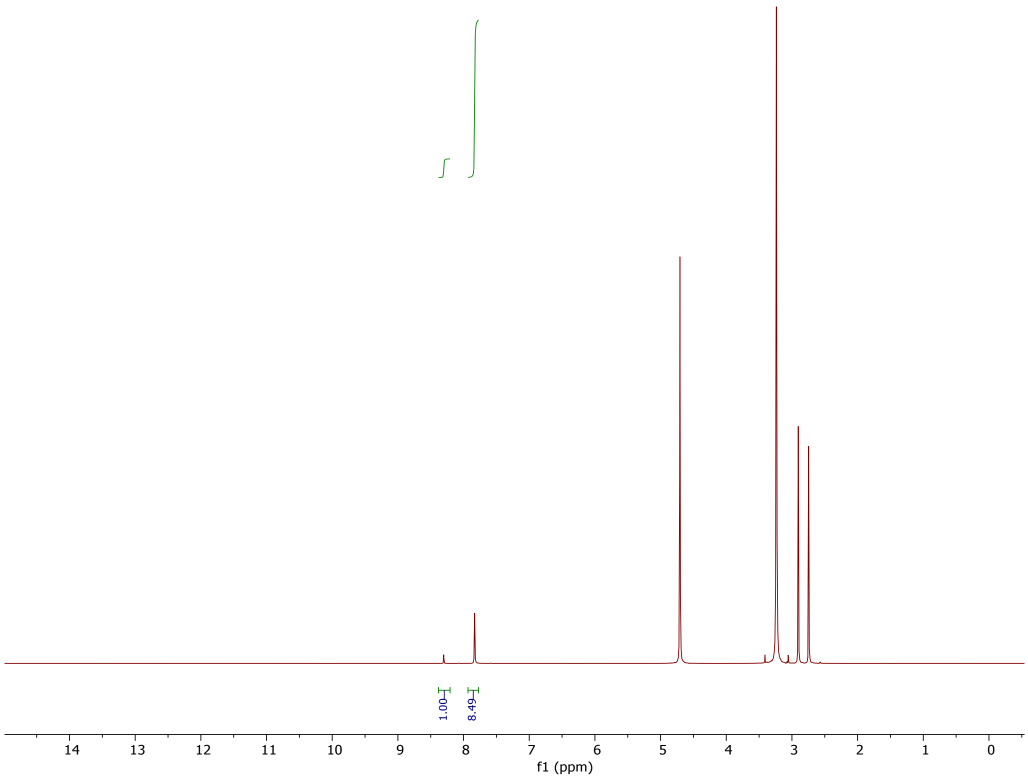

Figure S3. ^{1}H NMR spectrum for the reaction product obtained with D2-Ru-MOF catalyst.

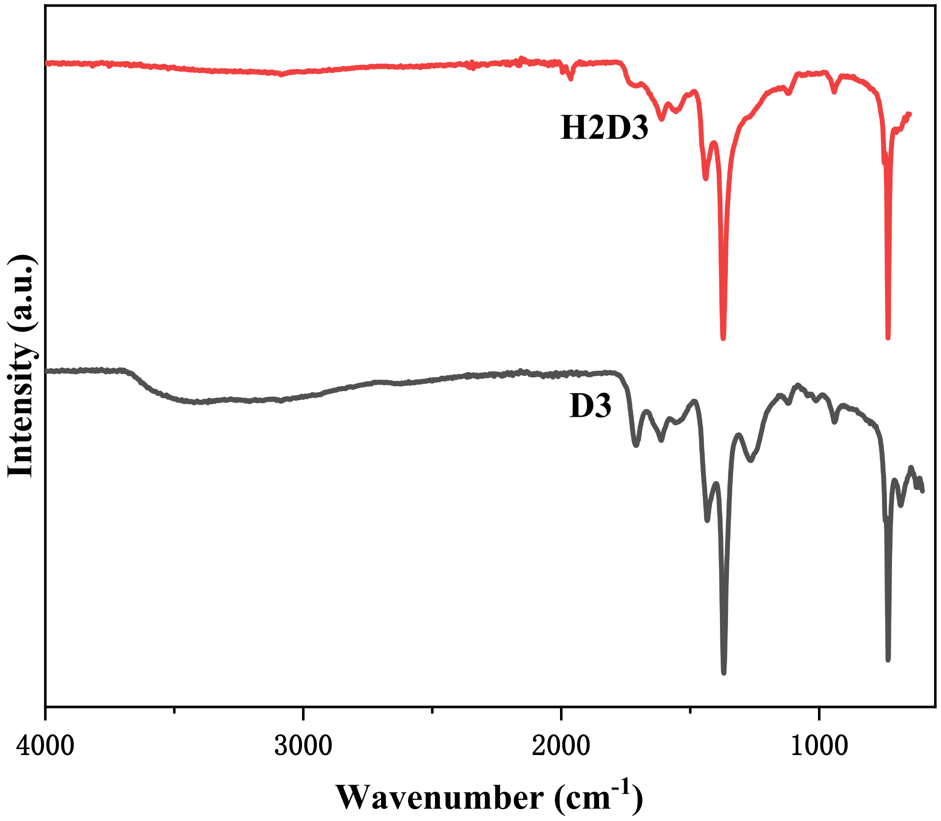

Figure S4. FTIR spectra of the D3-Ru-MOF catalyst before and after hydrogen treatment (H2D3-Ru-MOF).

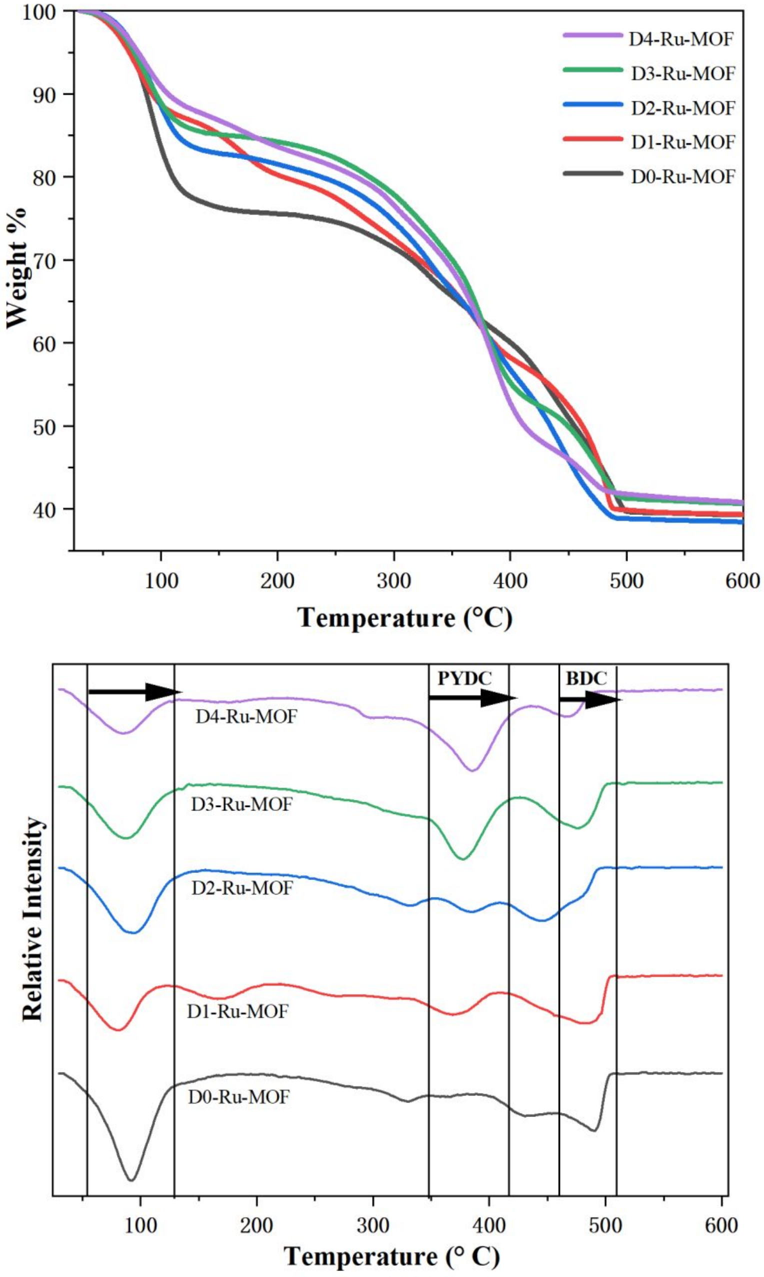

Figure S5. Thermogravimetric analysis (TGA) and derivative thermogravimetry analysis (DTA) curves of D0/D1/D2/D3/D4-Ru-MOF. Calculation of BTC and PYDC linkers in MOF structure.

(a) 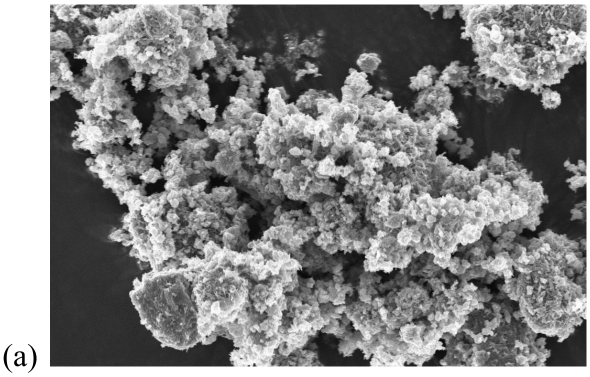 (b) 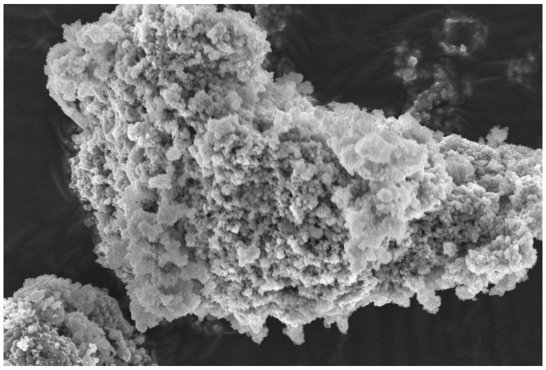

(c) 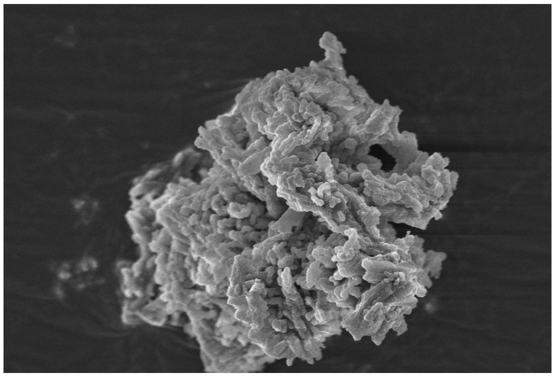

Figure S6. SEM images of D3-Ru-MOF catalysts: (a) before reaction, (b) after hydrogen pretreatment, (c) after the 4th reaction cycle.

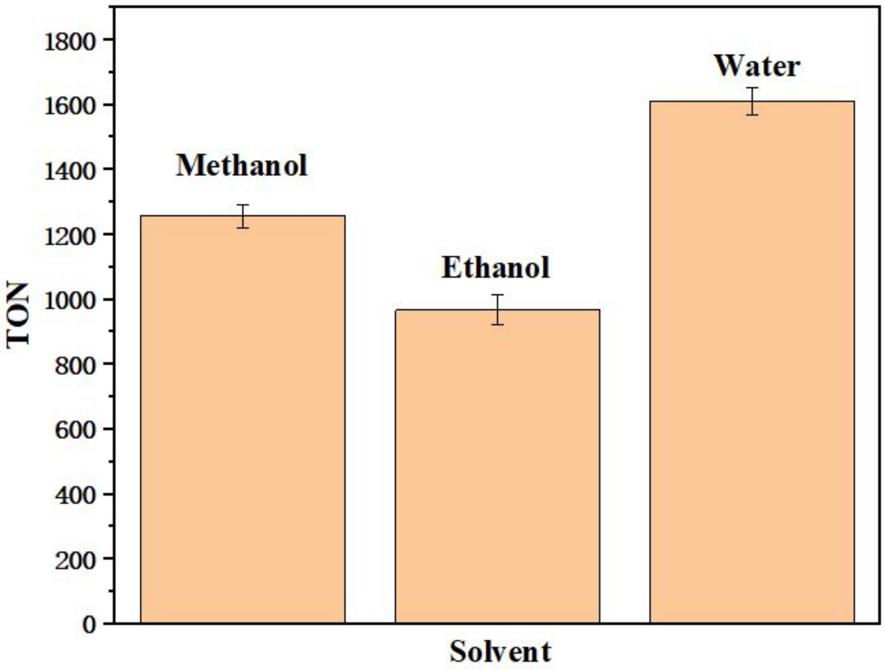

Figure S7. Effect of Solvent. Catalytic optimisation studies were conducted using 5 mg of H2D3-Ru-MOF, total solvent of 9.0 mL and 5 mmol KOH at 120 °C under a CO_2/H_2 (1:3) gas mixture with a total pressure of 60 bar. Each reaction was run for 24 h. * Although water affords the highest TON; the catalyst exhibits rapid deactivation after the first catalytic cycle when water is used as the reaction medium.

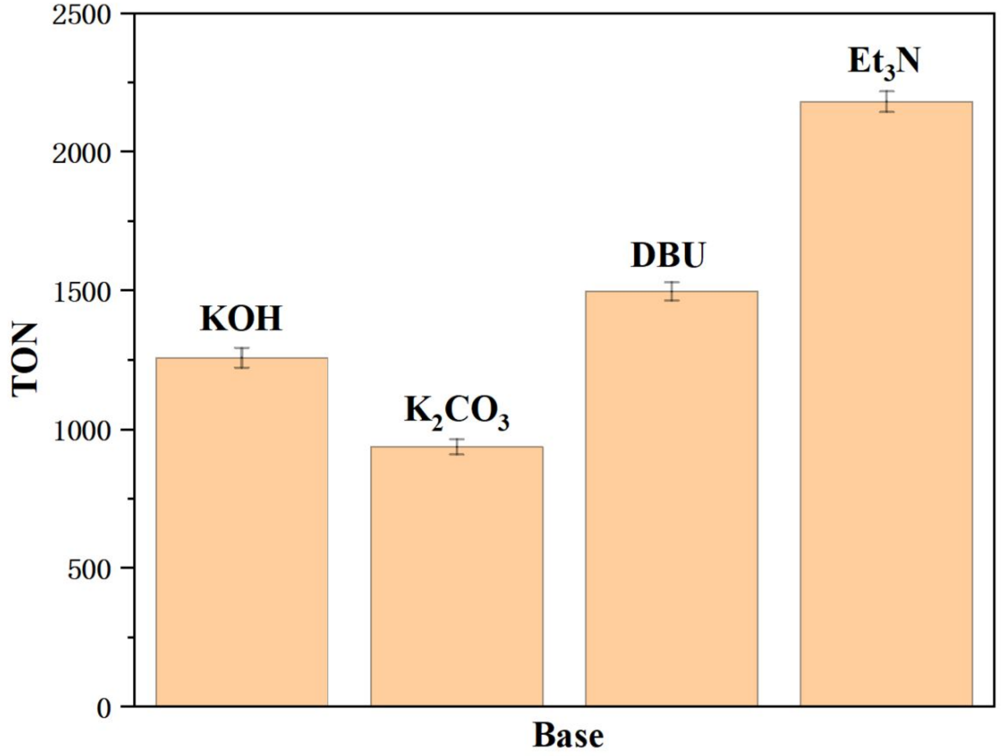

Figure S8. Effect of Base. Catalytic optimisation studies were conducted using 5 mg of H2D3-Ru-MOF, total solvent of 9.0 mL and 5 mmol base at 120 °C under a CO_2/H_2 (1:3) gas mixture with a total pressure of 60 bar. Each reaction was run for 24 h. * Although DBU and Et_3N affords the higher TON; the catalyst exhibits rapid deactivation after the first catalytic cycle when water is used as the reaction medium.

Table S1. Summary of results of studies using MOF as heterogeneous catalysts

MOF-Catalyst	TON	Ref.
[a]$RuCl_3$@MIL-101-NH_2-DPPB	831	1
[b]Ir(III)@JMS-5a	5474	2
[c]Rh(III)@JMS-5a	3257	2
[d]MOF1/Pd	1500	3

[a] P_T=6 MPa (CO_2/H_2 = 1:1, 120 °C, 2h, 25 mL DMSO+H_2O+PPh_3, Et_3N = 15 mL.

[b] P_T=6 MPa (CO_2/H_2 = 1:4, 110 °C, 24h, 8 mL EtOH, Et_3N = 0.7 mL.

[c] P_T=6 MPa (CO_2/H_2 = 1:4, 110 °C, 16h, 8 mL EtOH, Et_3N = 0.7 mL.

[d] P_T=3 MPa (CO_2/H_2 = 1:3, 170 °C, 2h, 8 mL THF, KOH = 5 mmol.

Table S2. Composition of Defective Ru-MOF catalysts with varying PYDC/BTC ratios and solvent content.

Catalyst	GM/Solvent %	Remove GM %	PYDC %	BTC %
D1-Ru-MOF	12	88	11.4	14.7
D2-Ru-MOF	16	84	15.5	9.8
D3-Ru-MOF	13	87	18.5	10.3
D4-Ru-MOF	11	89	11.2	5.3

Note: The temperature range for GM/Solvent%, PYDC% and BTC% is 53~129 °C, 348~416 °C and 456~503 °C. (GM represented for guest molecules)

Reference

(1) Wang, S.; Hou, S.; Wu, C.; Zhao, Y.; Ma, X. RuCl3 Anchored onto Post-Synthetic Modification MIL-101(Cr)-NH2 as Heterogeneous Catalyst for Hydrogenation of CO2 to Formic Acid. *Chinese Chem. Lett.* **2019**, *30* (2), 398–402. https://doi.org/10.1016/j.cclet.2018.06.021.

(2) Gumbo, M.; Makhubela, B. C. E.; Amombo Noa, F. M.; Öhrström, L.; Al-Maythalony, B.; Mehlana, G. Hydrogenation of Carbon Dioxide to Formate by Noble Metal Catalysts Supported on a Chemically Stable Lanthanum Rod-Metal-Organic Framework. *Inorg. Chem.* **2023**, *62* (23), 9077–9088. https://doi.org/10.1021/acs.inorgchem.3c00884.

(3) Makuve, N.; Darkwa, J.; Mehlana, G.; Makhubela, B. C. E. Hydrogenation of Carbon Dioxide to Formate Using a Cadmium-Based Metal–Organic Framework Impregnated with Nanoparticles. *Inorganics* **2022**, *10* (3), 30. https://doi.org/10.3390/inorganics10030030.
